# Commentary: Effectiveness of a hybrid digital substance abuse prevention approach combining e-learning and in-person class sessions

**DOI:** 10.3389/fdgth.2023.1158414

**Published:** 2023-11-02

**Authors:** Dennis M. Gorman

**Affiliations:** Department of Epidemiology & Biostatistics, School of Public Health, Texas A&M University, College Station, TX, United States

**Keywords:** drug prevention, selective outcome reporting bias, adolescent drug use, practical significance, study registration adherence

A Commentary on Effectiveness of a hybrid digital substance abuse prevention approach combining e-learning and in-person class sessions By Griffin KW, Williams C, Botvin CM, Sousa S, and Botvin GJ. (2023) Front. Digit. Health. 4:931276. doi: 10.3389/fdgth.2022.931276

This commentary contends that the results pertaining to substance use frequency reported in Griffin, Williams, Botvin, Sousa, and Botvin (2022) “Effectiveness of a hybrid digital substance abuse prevention approach combining e-Learning and in-person class sessions”, *Frontiers in Digital Health* are of no practical significance and are likely chance findings that emerge from the very liberal approach to statistical significance testing employed in the data analysis.

Griffin et al. conclude that their study “showed significant reductions in substance use” and that their results “demonstrate that substance abuse prevention programs conducted during middle school using hybrid e-learning modules plus a limited number of classroom sessions can produce meaningful reductions in substance use and improve important life skills” [(1), pp. 13−14]. These statements are based entirely on the statistically significant differences between the study conditions and take no account of the practical significance of the results.

**Figure 1:**
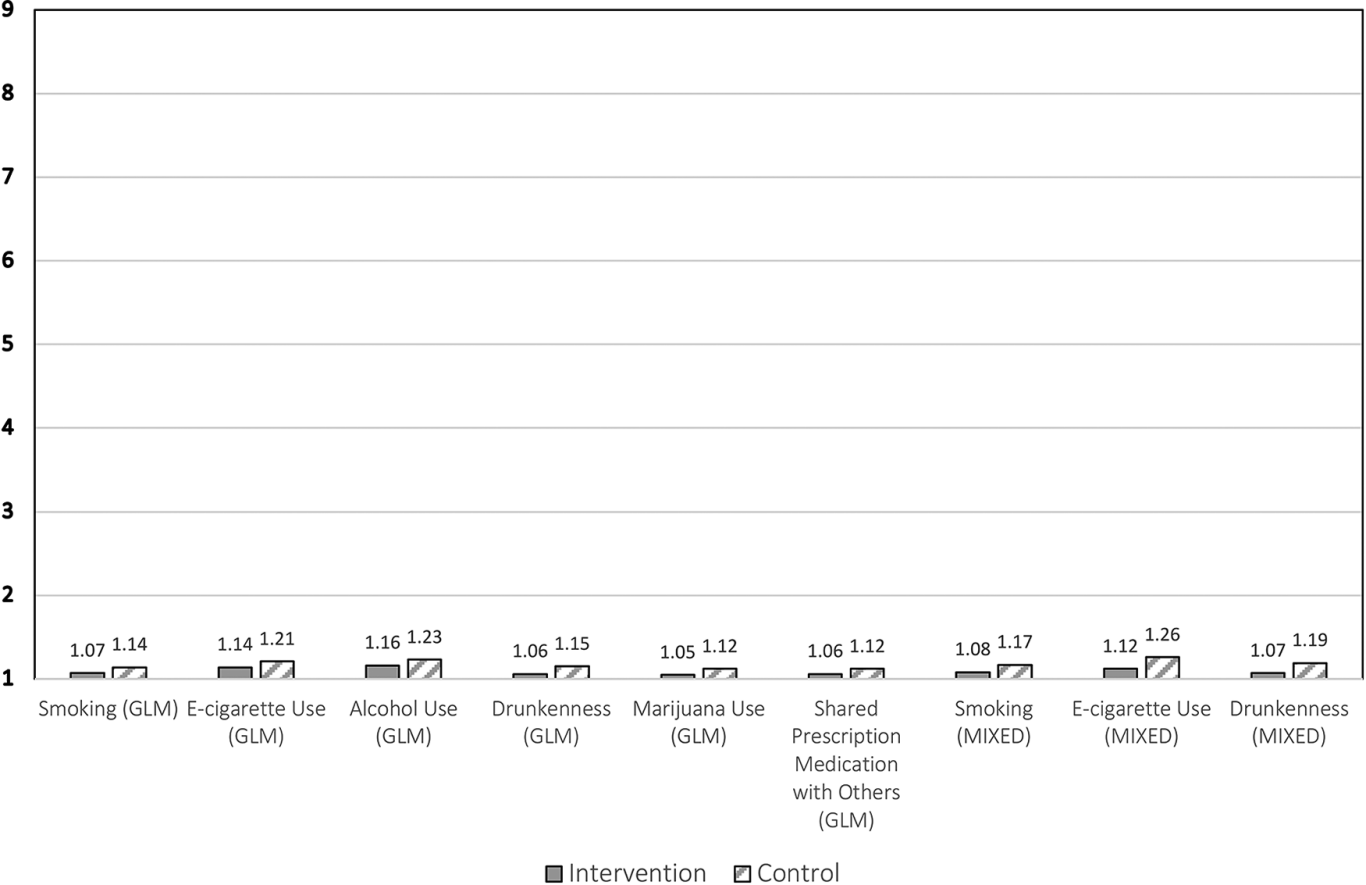
Adjusted post-test means on 9-point Likert scale for substance use outcomes that were statistically significant from the GLM and MIXED models.

Griffin et al. present two statistical models estimating the effects of the intervention, a GLM model and a MIXED model. The latter is the more rigorous as it accounts for clustering of students within the schools randomized to the two study conditions. Figure 1 presents the substance use outcomes for which a statistically significant difference was reported at post-test between the intervention and control groups using each model. It shows that the mean substance use of both groups on all outcomes occurs at the very low end of the 9-point Likert Scale, specifically between 1 (“Never”) and 2 (“A few times but NOT in the past year”). Given the age of the subjects, this is unsurprising. But, most noticeably, the differences between the two groups are very small on all outcomes: 0.06−0.09 for the six GLM model results and 0.09−0.14 for the three MIXED model results. The best interpretation of these results in terms of the scale used to assess the outcomes is that there was very little difference between the intervention and control groups at posttest, with both groups, on average, not having used these substances or shared prescription medication with others in the past year.

The analysis used by Griffin et al. is also bias towards finding statistically significant positive effects. As in most of their evaluations of the Life Skills Training (LST) program, they employ one-tailed tests of statistical significance. There is no justification for this, as at least one previous evaluation of the LST program found an iatrogenic effect on substance use (2). More generally, it is now widely recognized that the practice of associating statistically significant findings with *P *< .05 leads to a high false positive rate (3, 4). The potential for Type I error is made greater when all the value of alpha is allocated to a single side of the distribution, as is done with one-tailed tests of significance (5). Further, all but one of the results shown in Figure 1 is significant at *P *< .05. But since Griffin et al. do not report precise *P* values, as required by the American Psychological Association (6), one has no way of knowing if these very small differences would remain statistically significant had two-tailed tests been used. Thus, at a time when there is growing recognition that the threshold for statistical significance should be made more stringent and investigators refrain from simply reporting *P*-value thresholds, the evaluators of the LST program choose to conduct very lenient analyses that increase the chance of them producing false positive results.

The likelihood of false positive results appearing in Griffin et al.'s article is further increased by the number of statistical tests of significance conducted by the investigators. The results of the MIXED model for substance use outcomes are among 70 comparisons made between the study conditions in the paper. The best way to determine if these were the only statistical tests conducted by the investigators is through comparing the outcomes reported in the article to a document that prespecifies the outcomes of the study before data were collected and analyzed. The funding cited in Griffin et al.'s article is “National Institute on Drug Abuse, Grant Number: R44DA040358” [(1). p. 15]. This study was registered in ClinicalTrials.gov on April 26, 2017, with the title “Preventing Prescription Drug Abuse in Middle School Students (MSPDA)” (7). The primary outcome measure described in the registry is “Change in prescription drug use in the past year”, to be measured at posttest, 6-month follow-up, and 12-month follow-up. The only outcome related to prescription drug reported by Griffin et al. pertains to how frequently students shared prescription medications with others, and not change in students’ actual prescription drug use as described in ClinicalTrials.gov. No secondary outcomes, such as cigarette smoking, e-cigarette use, and drunkenness, are listed in the field provided for these in the registry. Also, according to the registry, the study concluded in June of 2020, so sufficient time has elapsed for the investigators to have analyzed the prescription drug use data from the 6- and 12-month follow-ups. In sum, like another recent registered evaluation of the LST program (8), the investigators do not adhere to the registered protocol when reporting the results of their evaluation.

Finally, Griffin et al. provide no information as to the extent to which the 622 subjects in the intervention group participated in the program, that is their level of involvement in the e-learning modules or the class sessions. In some of their previous evaluations, the investigators have emphasized the importance of implementation fidelity and participants receiving sufficient exposure to the program for it to be effective (9, 10), so providing no information about the delivery of this new hybrid version is a curious omission. While the effects of the program on substance use are of no practical significance and are likely false, chance findings, it would be useful to know how successful the program was in engaging the target population. One could then determine whether these findings were due to program failure or implementation failure.
